# Tuberculosis of Cattle*Lecture before the Pennsylvania Veterinary Medical Association at Allentown, Pa., Sept. 5th, 1892.

**Published:** 1892-10

**Authors:** Leonard Pearson

**Affiliations:** Veterinary Department, University of Pennsylvania


### TUBERCULOSIS OF CATTLE.*

By Leonard Pearson, B. S., V. M. D.

*Veterinary Department, University of Pennsylvania.*

The subject which has been selected for my talk to-day certainly has not the attraction of novelty. In some of its phases it is old and hackneyed, but other aspects have not been discussed in such minute detail as to render them tiresome.

It is useless to attempt to give an account of the causes, alterations, symptoms, etc., of tuberculosis, because these subjects are old to you and you are thoroughly familiar with them—perhaps too much so ; but we may well devote a few minutes of our time to some of the practical questions and recent developments in connection with this widespread disease.

When we have a case of colic or pneumonia to treat, we know exactly what to do : try to cure the animal as soon as possible. When we have a case of glanders or pleuro-pneumonia of cattle we also know exactly what to do : destroy the animal as soon as possible. But when a case of tuberculosis presents itself there is apt to be a wavering, a halting between two opinions. Numerous possible causes are open ; the animal, we will say, is productive and profitable, it is supplying a large quantity of milk or it is about to calve, or the owner is *almost* ready to send it to the butcher, or for some other reason wishes to keep it, and the decision of the veterinarian will, in many cases, be governed by the circumstances.

There is a greater lack of uniformity of action in dealing with this disease than is to be found in any other part of our professional duties. Some are in the habit of saying that a tuberculous cow is not dangerous until she becomes debilitated and manifestly sick. They ascribe tuberculosis to any one of a large number of debilitating influences and deny that the disease is contagious and that a tuberculous cow is a menace to the health of the herd.

Those who hold this view allow diseased animals to remain with their associates as long as they are profitable and then send them to the butcher. Others believe that a tuberculous animal is a dangerous animal, both to others of the same species and to man who consumes its products, and advocate the destruction of such animals as soon as the disease is diagnosed.

These represent, probably, the extreme views on this subject, but there are many who do not go to either limit and content them-

*Lecture before the Pennsylvania Veterinary Medical Association at Allentown, Pa., Sept. 5th, 1892.

selves with a modification of one opinion, or the other, depending upon their reading, observation, etc.

Let us review briefly, the ground that there is for saying that tuberculosis is contagious and that tuberculous animals (cattle particularly) are a source of danger to other animals and to human beings. Let us endeavor to find out if the upholders of this faith are guilty of exaggeration of the danger and of exciting undue alarm.

Those who are calling attention to the supposed dangers at present are not the first to give the alarm, for the laws of Moses forbid the use of meat of tuberculous animals, and it is also proscribed by the Talmud and the Semara (year 500 A. D.)

Church laws were made in Europe in the ninth century, which forbade the use of tuberculous meat and these were made more stringent or repassed in 1370, 1401, and 1558

In 1702 Florimus gave a good account of tuberculosis and named it the French disease, because it was supposed to be related to syphilis.

In the last century Germany had laws compelling the slaughter of all cattle suffering from tuberculosis, and the penalties for disregarding the law were very severe.

In 1783 it was shown that tuberculosis and syphilis were in no way dependent on one another, and the strict regulations governing the disposal of tuberculous cattle were relaxed. It is interesting to note that these laws were passed because it was thought that tuberculosis was a dangerous disease of itself, but they were annulled, because the secondary idea that tuberculosis and syphilis were related had become so firmly fixed in the public mind that when this view was disproved the other and formerly glaring dangers of tuberculosis were lost sight of and the necessity for the laws seemed to have disappeared. But history repeats itself, and laws relating to tuberculosis, similar to those formerly in force have already been passed in some places and other legislative bodies are discussing them.

That bovine tuberculosis was contagious found many advocates in the olden times, although there were more who ascribed the disease to climate, care, food, stabling, in breeding and various mysterious influences. In 1857 Buhl published, as his opinion, based on experiments, that tuberculosis is a specific infectious disease, and that it may be propagated by "tubercular virus" found in the lesions of the disease.

Villemin, in 1864, announced his theory that tuberculosis was a specific disease and caused only by the introduction into the body of the specific cause from without. This theory excited a vast amount of favorable and unfavorable criticism, countless experiments were made to test it, and the balance of opinion finally fell in Villemin's favor.

In 1882 Koch announced his discovery of the bacillus of tuberculosis, and all of the experiments made in the last ten years have but served to strengthen his statement that this bacillus is the sole exciting cause of tuberculosis. At present no experimental medical scientist denys or doubts the truth of Koch's statement.

The question of how the disease spreads is an interesting one. The bacillus may pass into the healthy animal by way of the lungs, digestive tract, sexual organs or skin, or may enter the unborn fœtus from the dam. The first method is the most common, although the others are by no means rare. Since the respiratory organs are most frequently first affected, the inference is natural and just, that the chief medium of infection is the air. The germs are inhaled. They reach the air from the lungs of diseased animals and from dried tubercular material from whatever source, i e.: mucous and pus from tubercular changes in the lungs, tubercular milk, tubercles from dead animals, etc.

If ventilation is poor and the cattle stand close together, the exhaled air from the diseased animals is not diluted by pure air, and is inhaled by other cattle in a comparatively concentrated condition, thus greatly increasing the danger of infection. Again, animals with a strong inherited predisposition require a slighter exciting cause to contract the disease, i. e., fewer bacilli, than cattle from untainted, vigorous parentage.

A case recently came under my observation in which three cows suffering with tuberculosis were found in a large herd quartered in a stable in which there were four rows of cattle. One of the three cows presented lesions, showing an advanced state of the disease, while the other two cases were more recent. All of these cows stood in the same row, and as the other cattle in the stable were healthy, the inference is natural that the two recent cases were due to their proximity to the more advanced case.

In another large herd it is clearly shown by the stable record that certain cows were veritable incubators of the disease, and that a place beside such an animal was almost as dangerous as a direct inoculation with tubercular material.

Reports are numerous of cases in which tuberculosis has been brought into a herd of healthy cattle by one diseased animal, and afterwards spread to a large percentage of the members of the herd.

Friedberger and Froehner say that many instances of this kind are known in which the disease spread from the newly added animal to the others *in order* until every animal in the stable became affected.

The distance to which the disease will spread from a diseased animal depends greatly upon the conditions, and in most cases, repeated exposure is necessary, and the nearer the healthy animal is to the source of infection the greater the danger. After a badly diseased cow has been removed from a stable, her stall and surroundings remain dangerous until thoroughly disinfected.

Inhalation experiments, in which the dust containing tubercle bacilli has been used as the infective agent, have been repeatedly made, and the disease tuberculosis set up in a large percentage of the cases.

That tuberculosis may be caused also by feeding on tuberculous material has been shown by direct experiment again and again, and observations have frequently been made in which the disease was conveyed, by this method, to animals and people consuming the products of tuberculous animals.

Klebs was the first (1868) to make feeding experiments with tubercular material, and succeded in propagating the disease in this way. He also called attention to the identity of bovine and human tuberculosis, and discouraged the use of milk from tuberculous animals, especially for children.

Chameau, in the same year, made a series of similar experiments, and arrived at the same conclusions reached by Klebs, and declared the flesh of tuberculous animals a dangerous food.

The feeding experiments of Gerlach, Günther, Harms, Zürn, Bollinger and Roloff, all show that the injection of tuberculous material is frequently followed by the development of the disease. All of these experiments were performed prior to 1876, but when, in that year, the German Advisory Board of Veterinarians was asked by the government whether the sale of tuberculous meat should be stopped they did not regard the question as settled, and did not give a definite answer. In the following years, however, the experiments and clinical observations were multiplied, and the proof became more positive that the disease is frequently transmitted by ingestion of diseased material, and cases in which this occurred under natural circumstances, both in man and animals, were reported.

The position taken a few years later by European veterinarians generally is shown by the report of a committee appointed to look into this matter by the International Veterinary Congress, at its meeting in Brussels in 1883. This report is chiefly the work of Lydtin, and contains among others the following declarations :

Tuberculosis is contagious to man as it is to animals.

There are clinical observations proving the transmission of tuberculosis, from animals to man, through the milk of phthisical animals.

The flesh and viscera of a tuberculous animal can only be utilized for consumption when the disease is found *in the cadaver* in its incipient stage, when the lesions are confined to a very small portion of the body, when the lymphatic glands are still free from all morbid tuberculous lesions, when the tuberculous formations have not yet undergone softening, when the flesh presents the character of meat of the first quality, and when the animal is in a good state of nutrition at the time of slaughter.

The question of the disposal of the milk from tuberculous animals was more summarily dealt with, for the resolution reads : "The milk of animals affected with tuberculosis or suspected of it, should not be taken by man nor by certain animals. The sale of this milk should be severely interdicted."

It has been claimed that such a measure is more stringent than necessary, for some maintain that the milk is not dangerous unless the udder contains tubercles. But the researches of Bang, Zschokke, Bollinger, Ernst and Peters and others, have shown that tubercle baccilli may be present in the milk when there are no traces of the disease in the udder, but the tubercles are situated in distant parts of the body.

In fact, this has become so well established, that in 1889 Friedberger and Froehner recommended the inoculation of guinea pigs with the milk of suspected cows as the surest means of diagnosing doubtful cases.

It is not rare to find tuberculosis in calves, and the lesions are far more common in the gastro-intestinal tract than in the respiratory organs, showing that infection took place through the first-named channel.

I have observed, also, that diarrhœa, tympany, and other digestive disturbances are much more common in calves in herds where tuberculosis is prevalent than among cattle free from this disease, which goes to show that the tubercle bacilli bearing milk is irritant and poisonous to the young animals. This observation has been previously made by Prof. Law and other veterinarians.

That similar changes may take place in infants consuming such milk is shown by the following statement by Dr. E. O. Shakespeare: "There is ample evidence that a large percentage of tuberculous milch cows produce milk which contains the infectious principle of this disease. The use of such milk for infant's food without boiling, constitutes an undoubted danger that they may become infected. In fact, since tuberculosis in the human race has become better known, it has been found that in infants and young children in some large cities the mortality from some form of tuberculosis is far greater than has been generally believed, amounting in some localities to one-fifth of the deaths of the young. The significant fact in this connection is that it is most frequently some part of the digestive passages that first become affected."

Another method by which tuberculosis is spread in herds is by copulation. Many cases have been described, and I saw one but recently in which the tubercle bacilli seemed to pass into the female through the sexual passages. That is, in these cases the diseases is either confined to the uterus and appendages, or is evidently primary in this organ. Bulls sometimes have tuberculosis of the penis and testicles.

That the disease may start in the skin is undoubted, but an abrasion is necessary for the multiplication of the germ, and this mode of infection is very rare.

The last means by which the disease is spread is heredity, and this was formerly thought to be the most important influence of all. But we now know that it is only the predisposition and not the disease which is inherited, and that tuberculosis does not appear until the germ enters the animal after its birth. In some exceptional cases animals are born with the disease, but, usually, when the fœtus becomes diseased the dam aborts. About 1,000,000 calves were killed in the slaughter house in Munich from 1878 to 1882, and of these but five had congenital tuberculosis.

But calves of tuberculous parentage—either on the side of the sire or of the dam—require less exposure and fewer influences favorable to the developments of the disease to cause it than calves from healthy parents.

In many cases in which heredity is supposed to play an important part, exposure after birth is the sole cause.

Prof. Law observes that calves may become infected by drinking the milk of a tuberbulous cow and the disease remain located for months when it spreads and may cause the death of the animal, in which case the the autopsy will reveal old calcified lesions together with the fresh ones.

From this discussion I think that it will be clear that tuberculosis is a contagious disease, the subjects of which are dangerous to men and animals, and we will be ready to agree with some of the preventive measures which have been advocated by different veterinanians and veterinary societies in Europe.

Prof. Johne of Dresden recommends: "That all tuberculous animals or those with tuberculosis tendencies must be unconditionally excluded from breeding.

"All animals diseased with tuberculosis must be separated from healthy ones and immediately slaughtered. Suspected ones should be treated in the same way.

"Stables in which such animals have been kept must be thoroughly cleansed and disinfected.

"Everything tending to cause a predisposition to disease must be carefully avoided, and great care given to ventilation, diet, exercise and exposure."

Similar measures are recommended by Veterinary societies and congresses everywhere.

It is evident then that the present haphazard method of dealing with tuberculosis should be abandoned and more strict and effective measures be substituted.

An effective plan would be to organize a regular inspection by veterinarians appointed by the State to discover cases of tuberculosis, and to order their slaughter.

This would, of course, necessitate the creation of a good many offices, as Assistant State Veterinarian, and the appropriation of a large sum of money for the use of the Secretary of the State Board of Agriculture to indemnify owners of condemned cattle.

But such an inspection is demanded to protect the health of the public, and to protect the healthy herds, and will save the State far more than it costs.

Although these measures have received the most powerful scientific support, there is a practical feature of the case which is frequently overlooked, but which has, in the past, rendered the administration of such measures most difficult and in many cases impossible.

It is well known that the symptoms of tuberculosis vary exceedingly with the location of the lesions, and that sometimes the disease may be quite severe, but so located that diagnosis by the ordinary methods is impossible. The tubercles may be situated in the liver, udder, intestines or lungs and not be discovered by a most careful physical examination. Then, auscultation of the lungs of cattle cannot be depended on to the extent to which we rely upon the results obtained by this method of examination in man, the horse or the dog. Sounds are heard, even in healthy lungs, which will deceive the most experienced ear, and slight lesions of the mucous membrane of the bronchial tubes may produce sounds similating those heard in pulmonary tuberculosis.

So when a disease appears in a herd and the evidently affected animals are removed, we have no certainity that there are not more subjects of tuberculosis remaining to scatter the germs broadcast and perpetuate the disease. In fact, this is the usual history of cases in which eradication is attempted. We have lacked a reasonbly sure means of diagnosis and this shortcoming has served to make it impossible to carry out most of the measures advocated to suppress the disease.

In Dec. 1890, Koch announced his discovery of a substance which had a specific action on tubercular processes, which, when properly administered, caused a hyperæmia around the tubercle, and an elevation of the body temperature. This action was not observed when the substance was injected into healthy men or animals or into subjects of diseases other than tuberculosis. This substance is known as Koch's lymph or tuberculin, and it was hoped, for a time, that it would cure consumption. But this hope has been abandoned.

In Jan., 1891, Prof. Gutmann of the Veterinary Institute of Dorpat, Russia, employed tuberculin on cattle and found that the results were quite similar to those obtained on man. That is, there was a temperature reaction in tuberculosis cattle and none in other cattle. The substance has since been used in the diagnosis of tuberculosis of cattle in every civilized land, and the reports are almost uniformly favorable. The Tuberculosis Commission of the University of Pennsylvania, of which Prof. Zuill was chairman, introduced its use for diagnostic purposes in this country, and the report of this commission was cautious, because the number of their experiments was limited, but it was decidedly favorable.

The Imperial Health office in Berlin has recently published an extensive report on the use of tuberculin in the diagnosis of tuberculosis of cattle which says that it has been found reliable in all sorts of cases, even when the lesion is hidden and the animal appears healthy, tuberculin reveals the presence of the disease.

The Berlin Veterinary Weekly, of which Prof. Dieckerhoff is the senior editor, contained an editorial in a recent number in which this statement occurred. "The proof which has been presented to our readers is more than sufficient. The results are absolute and gratifying, and show that tuberculin is a reliable agent for determining the presence of tuberculosis in cattle."

I have killed quite a number of cows which have reacted to tuberculin and have found them to be tuberculous in every case. I have seen other cows killed which were tested with tuberculin and did not react, and they were free from tuberculosis in every case, with but one exception. This exception was an old cow suffering from tuberculosis in a very bad form. She was given a small quantity of tuberculin and a typical reaction did not occur, but the tuberculin employed was old and the dose was small, and these circumstances, I think, account for the absence of the reaction.

That occurrences of this sort are rare is shown by this quotation from the editorial above mentioned: "When animals do not react after the injection of tuberculin it can be said with almost absolute certainty that they are free from tuberculosis; since not a single case has been unquestionably established in which animals containing tubercles have not reacted."

My results with tuberculin have been so satisfactory that I look upon its discovery as one of the most important advances which veterinary medicine has experienced in a number of years.

The method of using tuberculin is as follows: a 10 per cent. solution is made in a 1 per cent. solution of carbolic acid and of this dilution 2.5 to 5.0 cc. are injected beneath the disinfected skin of the scapular region. The injection had best be made about 6 P. M., at which time the temperature of the animal is measured. It is measured for 15 hours at intervals of 3 hours, and if an elevation beyond the nominal variation occurs, it is a reaction and the animal probably has tuberculosis.

The amount of reaction varies from 2 to 6 degrees, and lasts from 12 to 24 hours, or sometimes longer.

With this substance to aid us we are now in a position to practice what we preach, which we were not before, and can go on and enforce the regulations so long and earnestly advocated.

The danger is before us, it is among us, our mode of warfare is planned, our force and appliances are ready. All that is lacking is the legal authority.

